# Supplementary material for: Modelling atomic and nanoscale structure in the silicon–oxygen system through active machine learning
Source: Nat Commun. 2024 Mar 2;15:1927. doi: 10.1038/s41467-024-45840-9 (PMC10908788; doi:10.1038/s41467-024-45840-9)
Supplement: Supplementary file 1 — Supplementary Information [file 41467_2024_45840_MOESM1_ESM.pdf]

Supplementary Material for

“Modelling atomic and nanoscale structure in the silicon–oxygen system through active machine learning”

Linus C. Erhard,[1] Jochen Rohrer,[1,*] Karsten Albe,[1,†] and Volker L. Deringer[2,‡]

[1] *Institute of Materials Science, Technische Universität Darmstadt, Otto-Berndt-Strasse 3, D-64287 Darmstadt, Germany*

[2] *Department of Chemistry, Inorganic Chemistry Laboratory, University of Oxford, Oxford OX1 3QR, United Kingdom*

* rohrer@mm.tu-darmstadt.de

† albe@mm.tu-darmstadt.de

‡ volker.deringer@chem.ox.ac.uk

CONTENTS

Supplementary Note 1. Database 3

Supplementary Note 2. Calculation of physical properties 10

Supplementary Note 3. Performance of the ACE model 15

Supplementary Tables. 16

Supplementary Figures. 19

Supplementary References. 39

Supplementary Note 1. DATABASE

The composition of the database (DB) and the weights used for the fit are summarised in Supplementary Tab. I. The corresponding energy and force range can be seen in the scatter plot in Supplementary Figure 15. The DB contains a large fraction of structures used for training the SiO_2 GAP-22 [S1] (27%) and Si GAP-18 [S2] (22%) models. Structures added in the present work represent high-pressure SiO_2 phases (both crystalline and amorphous), surfaces, and charge-neutral vacancies, as well as Si/SiO_2 interfaces, quenched structures of Si–SiO_2 mixtures and clusters of $[SiO]_x$. These additional structures were partially designed on purpose and partially obtained by active learning (AL), either using small-scale models and the AL technique implemented in the MLIP package [S3] or using committee voting coupled to our amorphous matrix embedding, see **Supplementary Note 1** C 1. In both techniques we sample configuration space by molecular dynamics simulations (MD).

In the following, we provide details concerning the structure generation and related simulation parameters.

A. Manually prepared data

Most of the manually prepared data are based on crystal structures or other input structures with distorted unit cells and perturbed ("rattled") atomic positions. Unless stated differently, these structures have been prepared by applying variations of ± 2.5 % to the lattice parameters a, b, and c, and additionally by applying variations of ± 5 % to the cell angles α, β, and γ. Afterwards, the atoms were displaced using the `ASE` *rattle* function with a standard deviation of 0.01 Å.[S4]

1. Crystalline silica structures

We added distorted and rattled crystalline supercell models of the polymorphs α-quartz, β-quartz, moganite, coesite, stishovite, chabazite, α-cristobalite, β-cristobalite, low temperature tridymite, β-tridymite, and tridymite in the $C222_1$ and $P2_12_12_1$ modifications.

2. *fcc and hcp silicon*

We added snapshots of the fcc and hcp structures of silicon under high compression to the database to improve the repulsive behaviour of the potential at short interatomic distances.

3. *High-pressure silica*

We included simple unit cells, as well as $2 \times 2 \times 2$ supercell expansions, of pyrite-type and α-PbO_2-type silica in our database.

4. *Silica surfaces*

Supplementary Table II shows a list of surfaces which we included into our database. For each of these surfaces we included 30 structural models in the database, of which 10 were obtained by rattling the structures with an average displacement of 0.01 Å, 10 with an average displacement of 0.05 Å, and 10 with 0.1 Å. Additionally, we modified the surface terminations of several of these structures, for each creating 10 structural models and rattling each of those by 0.01 Å. Amorphous surface-structure models were created by using amorphous bulk samples and cleaving them to create an (artificial) open surface.

5. *Silica vacancies*

Vacancy structures were created by randomly removing atoms corresponding to one formula unit of SiO_2 from α-quartz and amorphous structure models.

6. *Crystalline–amorphous Si–SiO_2 interfaces*

Crystalline–amorphous interfaces between silicon and silica were modelled by merging two crystalline bulk supercells together. One of these supercells was kept fixed, while the other was melted. A repulsive wall was used to prevent atoms from the molten phase to diffuse into the crystalline phase.

7. *Clusters*

$[SiO]_x$ clusters were generated by iteratively performing MD simulations. We started with an $[SiO]_2$ cluster and added five snapshots from five MD simulations (total of 25) to the database. Within these MD simulations, the temperature was increased from 100 to 2000 K. After adding the structures to the database, we refitted the potential and repeated the simulations with clusters containing one additional formula unit of SiO, resulting in an $[SiO]_{i+1}$ cluster. We repeated the process up to a system size of $[SiO]_{32}$.

B. Small-scale active learning

In the small-scale active learning part of the work, we used tools implemented in the `MLIP` package.[S3] We performed MD simulations and recognised new structure using the maxvol algorithm as implemented in that code. We used an extrapolation threshold of 1.5 and a stopping threshold of 3.0. This active learning process was repeated iteratively. We performed about 100 simulations in parallel, collected newly found structures and reduced them to the most relevant ones by using the maxvol algorithm. Then we computed the corresponding energies and forces using DFT, refitted the potential, and repeated the MD simulations.

In the following, we briefly give the simulation details of the performed MD simulations. We performed quenching simulations under various conditions. All these simulations start with a randomisation phase for 10 to 100 ps at 6000 K and under NVT conditions. This is followed by equilibration at 4,000 K under NPT conditions for 100 ps and subsequent quenching to room temperature with various rates (10^{12} to 10^{14} K/s).

1. *Quenching under ambient pressure (SiO_2 and SiO_x)*

We performed quenches with the SiO_2 compositions and mixed SiO_x compositions under ambient pressure using the quenching procedure explained above (external pressure of 0 GPa). For the SiO_2 simulations, we used various crystalline unit-cell and supercell models as input. For the mixed simulations, we stacked together various SiO_2 and Si crystalline cells to achieve different compositions.

2. *High-pressure amorphous silica*

Similar to the ambient pressure simulations, we also performed quenching simulations for the high-pressure phases. However, this time we used pressures between 0–200 GPa in the NPT part. After convergence for quenching simulations (*i.e.*, once the active-learning approach did not identify new structures any more), we performed compression simulations using amorphous input structures under NPT conditions, starting from a pressure of 0 GPa going to a pressure of 200 GPa. The temperature was held constant at a random value between 0 and 1000 K.

3. *SiO_2 surfaces*

We used the manually created surface structures described above as input for MD simulations. We annealed the surfaces under NVT conditions from 50 K to 3000 K and cooled them back down to 50 K afterwards.

C. Large-scale active learning

In the large-scale MD simulations we used a committee error to examine the uncertainty of each atom. Based on the committee error, we decide whether a structure needs to be added to the database. In the case that we want to add the structure, we extract a small-scale model by amorphous matrix embedding. In this section, we briefly explain the amorphous matrix embedding method and then give the details for the MD simulations.

1. *Amorphous matrix embedding*

Amorphous matrix embedding is used to enable active learning (AL) for large-scale atomistic systems. For DFT calculations, system sizes are typically restricted to a few hundreds of atoms. Therefore, conventional AL is performed using models of this size. During large-scale simulations, however, local atomic environments may partially deviate from environments encountered in small-scale simulations. It is therefore desirable to include environments that are likely to appear only in (very-) large-scale simulations. Amorphous matrix embedding is a technique that allows us to do so.

The basic idea of the amorphous matrix embedding technique is to locally identify unknown environments in a large-scale simulation, extract this environment including its surrounding (a total of 200–400 atoms), freeze the atoms in the relevant environment and amorphise the surrounding using periodic boundary conditions. This idea is sketched in Fig. 1b in the main paper. As a measure for the uncertainty of an environment, we use committee voting of several moment-tensor potentials (MTPs) with respect to forces on a particular atom. In particular, we define the uncertainty u_α as

$$u_\alpha = \sqrt{\sum_{i \in x,y,z} \sigma(F_{\alpha,i}^{(1..N)})^2}. \tag{S1}$$

Here, $F_{\alpha,i}^{(1..N)}$ is the i-th component of the force acting on atom α as obtained by one of the N committee members (potentials) for a particular configuration along a trajectory; σ denotes the standard deviation.

Based on this, the amorphous matrix embedding is performed now according to the following procedure. First, we loop over all atoms and find atoms with an uncertainty above a certain threshold (1–2 eV/Å). In principle, this indicates that the local environment of the atom is unknown to the potential. However, if the uncertainty is very high, the structure might be very unfavourable and contain atoms with very high forces. This would worsen the DFT convergence and, subsequently, the potential fit. Therefore, we introduce an upper threshold (5 eV/Å), which is not limited to the atom itself, but also to atoms in the direct environment. If one of the atoms exceeds that threshold, we do not use this environment. Another issue is that similar structures might be picked in subsequent steps of the MD trajectory. We use smooth overlap of atomic positions (SOAP) similarities as implemented in `DScribe`[S5] to add only structures, which are not similar to previously selected atoms (similarity $<$ 0.9–0.95).

Finally, for atomic environments that fulfil all the requirements, we create a new box with a box size of 13 Å plus an additional margin of 1 Å. The reason for this box size is that it is larger than twice the cutoff while still being DFT feasible. We cut out a cube with a side length of 13 Å around the atom of interest and fill this cube into the newly created box. Depending on the scenario, the composition of the box is adjusted by deleting atoms outside of the region of interest. To fix the artificial boundaries we annealed the atoms outside of the cutoff up to 2000 K to 6000 K while keeping the inner atoms fixed. Afterwards, the structures were quenched to 300 K. To prevent atoms from outside the cutoff to enter the

cutoff area, we applied a repulsive potential centred around the atom of interest.

For surfaces, further special adjustments had to be made. If the atom was close to the surface, we estimated the normal vector of the surface by

$$\mathbf{n}_i \approx \sum_j (\mathbf{x}_i - \mathbf{x}_j) \text{ if distance}(i,j) < \text{cutoff}, \tag{S2}$$

where $\mathbf{x}_j$ is the position of atom j. The box to be extracted was then rotated in a way that the normal vector was oriented along the z-direction. Additional vacuum layers of 5 Å along each direction were placed into the direction of the normal vector. After the MD simulations, some of the surface structures had not been periodically connected into the x and y directions. These cases were handled as cluster (rather than slab) structures, and additional vacuum in the x- and y-directions was added, to be able to use the dipole correction in VASP.

2. *Quenching at ambient pressure (SiO_2 and SiO_x)*

We used the same protocol for quenches as mentioned in the small-scale active learning part. As input structure for the SiO_2 simulations, we used β-cristobalite supercells, while for the SiO_x simulations we used supercells of the cell used for the small-scale simulations. For the latter case, an exemplary structure is shown in Supplementary Fig. 16.

3. *Silica surfaces*

To perform large-scale active learning, we used the same standard quenching protocol as mentioned above. However, the NPT part was replaced by a part, where the initially liquid structure was constantly strained to larger volumes inducing pores into the structure.

4. *Vacancies*

As input structures, we used quartz supercells and amorphous structures containing about 65,000 atoms, including randomly selected vacancies corresponding to 50 formula units. These structures were heated from room temperature to 3000 K and cooled back to room temperature in MD simulations.

5. Compression to very high pressures

We used amorphous models containing 65,000 atoms, which we compressed up to 200 GPa in MD simulations, at a constant randomly selected temperature between 0 and 1000 K.

6. Clusters

We used a box containing 10,000 SiO molecules in the gaseous state with a density of 0.011 g/cm^3 as input structure. This box was compressed to a density of $\approx$ 2 g/cm^3, while keeping the temperature constant at 1,400 K.

Supplementary Note 2. CALCULATION OF PHYSICAL PROPERTIES

A. Phase diagram calculation

For the determination of the phase diagram we used calphy [S6], which implements thermodynamic integration using reversible scaling [S7] and nonequilibrium calculations of free energy differences. The basic idea is that we are switching the Hamiltonian of our system continuously to the Hamiltonian of a reference system with known free energy. Calphy uses either the Einstein crystal for crystalline structures or the Uhlenbeck–Ford model [S8] for liquid systems. Finally, we obtain an absolute Gibbs free energy for a certain temperature and pressure.

This Gibbs free energy is used as a reference energy, from which we start to perform thermodynamic integration using reversible scaling. By doing so, we are able to find the temperature dependence of the Gibbs free energy for a given pressure or (although not used here) also the pressure dependence for a given temperature. Details of the theoretical background can be found in the corresponding publication.[S6]

We used input structures with a cell size of around ≈ 15,000 atoms. For the calculations we used 50,000 equilibration steps and 800,000 switching steps for switching between our systems and the corresponding reference systems. For the thermodynamic integration, we used 300 steps/K. The force constants for the Einstein crystal were set to 2 eV/Å^2 for oxygen and 4 eV/Å^2 for silicon to avoid numerical instabilities. We carefully checked the dependence of the final results on the force constants and can exclude notable impact (ΔG <0.01 meV/atom).

In our work, we sampled the free energies in a temperature range between 600 K and ∼2,600–3,000 K for various polymorphs. An example is shown in Supplementary Fig. 17a. Here, we show the Gibbs free energies for four different structure types of silica at a pressure of 0 GPa. For cristobalite we started integration at 1,500 K up to shortly above the melting point. The tridymite interval was split into two integrations intervals to achieve higher accuracy. One integration was performed from 1,500 K to 600 K and the other from 1,500 K to 2,600 K. The melt was integrated down from 4,000 K to 1,500 K.

We cannot integrate quartz in the same way since α-quartz is dynamically switching to β-quartz in the simulations. Therefore, the α-quartz / β-quartz transition was determined

using classical MD simulations (driven by the ML potential). This is illustrated in Supplementary Fig. 18a–b. α-quartz as well as β-quartz input structures were equilibrated in MD simulations at various temperatures for 100 ps. Already around 30 K away from the transition points the final densities agree very well. The transition temperature was determined by finding the maximum slope of the following approximating function,

$$\rho(T) \approx \begin{cases} a \cdot x^2 + b \cdot x + c & x < T_1 \\ 0.5 \cdot \cos\left(\dfrac{x - T_1}{T_2 - T_1}\pi\right) + 0.5 & T_1 < x < T_2 \\ d \cdot x + e & x > T_2 \end{cases} . \tag{S3}$$

The parameters a, b, c, d, e, T_1, and T_2 were fitted to the results from the MD simulations. This process was repeated for several pressures. Corresponding transition temperatures in dependence of the pressure are shown in Supplementary Fig. 18c. A line is shown, which was fitted to the average transition temperatures. This line was also used in the phase-diagram plots in Fig. 2 of the main text. Based on this transition line, we performed thermodynamic integration for α-quartz from 600 K to 50 K below the α–β-quartz transition line, while for β-quartz we started at 50 K above this line. Therefore, we do not have data for quartz in a range of 100 K around the transition line.

This whole process was repeated for several pressures, as indicated in Supplementary Fig. 17b for α-quartz. Corresponding to the pressure, the temperature integration interval was adjusted for each polymorph. Finally, to calculate the phase diagram, we fitted our data points to a polynomial of third order to achieve an equation for the Gibbs free energy dependence of the pressure. This was done for temperatures between 600 and 2900 K in 1 K steps. Exemplary, this is shown in Supplementary Fig. 17 for α-quartz for temperatures of 650, 1000, 1500 K.

Supplementary Fig. 1 emphasises the difficulties of achieving accurate agreement between calculated and measured phase diagrams. We show how decreasing the stability of certain phases changes the phase diagram compared to the original one. The original phase diagram is shown as a thin red line in the background, while the modified one is shown by black lines. For quartz, coesite, and the melt, we added 2 meV/atom to the free energy since the coexistence lines are less sensitive to a shift of the energy compared to cristobalite and tridymite (for which we added only 0.1 meV/atom). These results are shown in Supplementary Fig. 1d–f. The corresponding change of the transition lines is shown in Supplementary

Fig. 1b–c. Additionally, in Supplementary Fig. 1a we show how the phase diagram changes when we add 5 meV/atom to cristobalite and tridymite. The additional blue line in the background shows the CALPHAD reference data from [S9].

There are several points we can conclude from this. First, there is a high uncertainty in the determination of the cristobalite–tridymite transition line, especially with respect to the error of potential compared to the corresponding DFT data (≈1 meV/atom). Second, it shows how essential the choice of the DFT exchange–correlation functional is, since depending on the functional this phase diagram would look very different. The reason for this is that the energetic differences of silica polymorphs, if predicted by different exchange–correlation functionals, can vary by more than 100 meV/atom.[S1] Thirdly, we can see that also the SCAN exchange–correlation functional is not "perfect": destabilising cristobalite and tridymite would improve the match with the experimental phase diagram significantly. Therefore, it seems like that the SCAN energies for these polymorphs are predicted slightly too low.

Another point is that the melting point is significantly overestimated by the ML potential at low pressures. This can, in principle, be due to two reasons, namely the exchange–correlation functional and an undersampling of the liquid phase space. The latter would likely correspond to a high error in the prediction of the energy of the liquid phase. To assess this, we analyse the 3000 K ACE-MD test set (see Supplementary Table III) for the liquid phase, which has an energy RMSE error of 3.7 meV/atom. When we assume that the energy of the liquid phase is overestimated by this value by the potential, and correct the phase diagram accordingly, we obtain the phase diagram shown in Supplementary Fig. 19. In this phase diagram, the melting point is still overestimated by 260 K, which thus indicates that (at least partially) the exchange-correlation functional is likely the reason for the deviation of the predicted melting point from experiment.

B. Enthalpy and structural analysis at high pressure

Figure 3 in the main text shows the enthalpy, H, for several phases. The enthalpy is given by

$$H(p) = E(V) + p(V) \cdot V, \tag{S4}$$

where E is the internal energy, p is the pressure, and V is the volume. The volume

dependence of the energy was determined by a Birch–Murnaghan fit to the energy–volume curve of each polymorph. $p(V)$ was given by the corresponding derivative. The energy-volume curves were calculated by varying the volume by ± 20 % for α-quartz and coesite, by ± 25 % for stishovite and ± 30 % for all other phases. The corresponding structures were structurally optimised, allowing changes of the positions as well of the box shape, however keeping the volume fixed.

For the analysis of the compression MD simulations, coordination numbers were determined by integrating over the first peak of the partial Si–O radial distribution function. The Si–O bond distances are given by the first peak position of the partial Si–O radial-distribution function.

C. Thermal expansion coefficient

Supplementary Fig. 4 shows the thermal expansion coefficient of diamond-type silicon, computed within the quasi-harmonic approximation [S10] as implemented in `phonopy`. [S11,12]

D. Amorphous silicon

Supplementary Fig. 5 shows the structure factors of two amorphous silicon structures. One was taken from ref. 13 (generated using the GAP-18 model [S2] in that work) and one generated in the present work by using the ACE model, a quench rate of 10^{11} K/s, and a system size of 8,000 silicon atoms. The structure was randomised at 2,500 K followed by holding for 100 ps at 2,000 K. Afterwards, the structure was quenched to 500 K and then held at 300 K for 20 ps to determine the structure factor.

E. Interface energies

Interface energies as in Supplementary Fig. 10 are calculated according to

$$\gamma = \frac{E_{\text{structure}} - N_{\text{SiO}_2} \cdot E_{\text{ref}}^{\text{SiO}_2} - N_{\text{Si}} \cdot E_{\text{ref}}^{\text{Si}}}{A}, \tag{S5}$$

where $E_{\text{structure}}$ is the energy of the whole structure containing the interface, A is the total interface area, $E_{\text{ref}}^{\text{SiO}_2}$ is the reference energy of the silica part of the structure, and $E_{\text{ref}}^{\text{Si}}$ is the reference energy of the silicon part of the structure. For the small-scale models, reference

energies are given by the input structures for the interface constructions. Their atomic positions are relaxed using the ACE model. In the case of DFT energy values, we carried out single-point computations for the ACE-relaxed structures.

F. Interface energy model for the SiO crystallisation

In this section, we will show several results for computed interface energies for large-scale manually constructed interface models and compare these to the interface energies of the SiO models. Moreover, we will create a simple model to understand when it is favourable for silicon to crystallise within an SiO matrix.

Using Eq. S5, we calculated interface energies for different combinations of manually constructed interfaces between crystalline and amorphous silicon as well as amorphous silica. The energies of the structures used to build these interfaces are given in Supplementary Table IV. Exemplary interface models are shown in Supplementary Fig. 13a-c. These structure are constructed from amorphous sample structures, which have been created with various quench rates (1×10^{13}, 5×10^{12}, 2×10^{12}, 1×10^{12} K/s). After inserting a spherical inclusion into the matrix, the structures are optimised at 0 K, heated from 10 K to 300 K within 50 ps, held there for another 50 ps and then quenched to 10 K within 50 ps. The structure is then relaxed again. The whole process is carried out under NVT conditions.

Supplementary Fig. 13d shows that the interface energy does not change much with the input structure. However, this does not mean that this is the "real" interface energy between both phases. This becomes clear when we compare the SiO interface energy, which is actually an a-Si–a-SiO_2 interface, with the interface energy of the manually constructed a-Si–a-SiO_2 interface. The latter is significantly higher. This could be explained by two reasons, namely, interface relaxations and interface orientations. During the quench MD simulation it is, in principle, possible for the atoms to arrange in such a way that only favourable interface orientations occur. In contrast, in the hand-built model, more and less favourable orientations might occur. Moreover, during the quench the interface atoms are able to relax properly, whereas in our simulation setup this was not always possible.

Supplementary Note 3. PERFORMANCE OF THE ACE MODEL

Supplementary Table III shows a more comprehensive version of Table 1 in the main text. The table shows that in nearly all cases, the accuracy of the complex ACE potential is better or comparable to the accuracy of our previously reported SiO_2-GAP-22 model. [S1] Indeed, the forces are significantly more accurate, while the difference for the energy is not too large. Similarly, Supplementary Table V shows that the ACE model is at the same time more than 150 times faster than the GAP. Moreover, the speed is only slightly lower compared to the linear and Finnis–Sinclair-like ACE models.

Supplementary Tables.

Supplementary Table I. **Composition of the total database** subdivided in various structure types with various compositions. We also indicate which parts of the database are taken from previous work and which parts are new. For the structures collected by active learning, we indicate which active learning approach was used ("small-scale" or "large-scale" as discussed in the text). We also give the fitting weights for each of the structure types.

Configuration type	Composition	Reference	Active learning	Structures	Atoms	Weights
crystalline	SiO_2	SiO_2 GAP + this work	—	2,620	281,820	100
amorphous	SiO_2	SiO_2 GAP	—	313	60,096	1
half-quenched	SiO_2	SiO_2 GAP	—	311	59,712	1
liquid	SiO_2	SiO_2 GAP	—	313	60,096	1
crystalline (main)	Si	Si GAP	—	1,257	38,680	100
amorphous	Si	Si GAP	—	159	29,632	1
liquid	Si	Si GAP	—	76	5,312	1
surfaces	Si	Si GAP	—	214	22,066	1
defects	Si	Si GAP	—	423	74,548	1
various (e.g. high energy crystal)	Si	Si GAP + this work	—	505	2,556	1
quenched	SiO_2	this work	small-scale	385	19,008	1
quenched	SiO_2	this work	large-scale	417	53,208	1
vacancies	SiO_2	this work	—	278	56,520	1
vacancies	SiO_2	this work	large-scale	780	121,836	1
high-pressure crystals	SiO_2	this work	—	400	19,080	1
high-pressure amorphous	SiO_2	this work	small-scale	166	31,872	1
high-pressure amorphous	SiO_2	this work	large-scale	407	120,246	1
surfaces	SiO_2	this work	—	603	48,477	1
surfaces	SiO_2	this work	small-scale	28	1872	1
surfaces	SiO_2	this work	large-scale	167	8466	1
crystalline-amorphous interfaces	Si+SiO_2	this work	—	457	31036	1
quenched	Si+SiO_2	this work	small-scale	457	31036	1
quenched	Si+SiO_2	this work	large-scale	430	71821	1
clusters (dimers, larger SiO clusters)	various	SiO_2 GAP + this work	—/large-scale	611	24,900	1

Supplementary Table II. **Crystalline structures with surface orientations** which are included in the database.

Polymorph	Surfaces
α-quartz	(001), (110), (100), (210)
α-cristobalite	(001), (100), (110)
low temperature tridymite	(100), (001)
β-tridymite	(001), (110), (100), (210)
β-cristobalite	(100), (110), (111)
moganite	(100), (010), (001)

Supplementary Table III. **Comparison of the accuracy** of the SiO_2-GAP-22 model[S1] with various ACE models for the training and testing set, as well for a range of separate smaller datasets. The errors given in the table are root mean square errors for energies and forces. The unit of the energy error is meV/atom and the unit of the force error is meV/Å.

				SiO_2-GAP (Ref. 1)		Si–O-ACE (This work)					
						Linear ($N=1$)		F–S ($N=2$)		Complex ($N=8$)	
	Details	$N_{\text{structures}}$	N_{atoms}	ΔE	ΔF	ΔE	ΔF	ΔE	ΔF	ΔE	ΔF
Training		10853	1192984	—	—	70.7	492	31.5	436	17.7	306
Testing		571	65208	—	—	51.5	490	33.1	431	16.7	305
SiO_2 crystals	α-quartz,coesite,stishovite	15	840	1.0	82	0.8	74	1.1	62	0.9	45
SiO_2 surfaces	amorphous	5	540	14.9	178	21.4	206	18.0	182	4.7	156
a-SiO_2 (ACE-MD)	T=500,1500,3000 K	15	2880	4.0	173	8.0	277	7.4	260	3.2	176
a-SiO_2 (CHIK-MD)	T=300 K, see Ref. 1	5	960	3.7	188	4.1	270	5.1	267	2.2	192
a-SiO_2 (GAP-MD)	T=300 K, see Ref. 1	5	960	1.1	101	10.3	132	9.8	120	4.6	96
a-SiO_2 (BKS-MD)	T=300 K, see Ref. 1	5	960	1.7	130	3.3	210	3.2	185	1.3	132
a-SiO_2 (Vashishta-MD)	T=300 K, see Ref. 1	5	960	5.7	221	9.9	330	10.0	325	3.1	224
a-SiO_2 (Munetoh-MD)	T=300 K, see Ref. 1	5	960	8.5	508	75.9	591	55.3	552	22.0	371
Amorphous Si	T=500,1500,3000 K	15	1920	> 1600	> 3200	115.8	375	53.9	339	51.5	258
Mixed phases	T=500,1500,3000 K	15	1920	> 4200	> 3500	37.8	710	35.0	635	38.0	431
High pressure	p=50,150 GPa	10	1920	122.7	873	15.1	476	5.6	359	4.6	236

Supplementary Table IV. **Reference energies and volumes for interface calculations.** Energies of crystalline structures (diamond and α-quartz) using the ACE potential, as well as of various amorphous structures generated by different quench rates, are given.

	Energies	
Type	silicon (eV/atom)	silica (eV/SiO_2)
crystalline (ground state)	–5.8633	–26.0359
amorphous (1×10^{12} K/s)	–5.6809	–25.8543
amorphous (2×10^{12} K/s)	–5.6740	–25.8462
amorphous (5×10^{12} K/s)	–5.6644	–25.8312
amorphous (1×10^{13} K/s)	–5.6548	–25.8154
	Volumes	
Type	silicon (Å^3/atom)	silica (Å^3/SiO_2)
crystalline (ground state)	20.0003	37.5105
amorphous (1×10^{12} K/s)	20.2170	45.6340
amorphous (2×10^{12} K/s)	20.2263	45.5906
amorphous (5×10^{12} K/s)	20.2138	45.5631
amorphous (1×10^{13} K/s)	20.1974	45.4809

Supplementary Table V. **Timing and speedup** of various ACE potentials compared to our GAP model from Ref. 1. Timings were obtained for 192 atoms cells over 100 time steps.

	GAP (Ref. 1)	ACE (This work)		
		Linear ($N = 1$)	F–S ($N = 2$)	Complex ($N = 8$)
Timing (μs/(timestep $\cdot$ atom))	11,037	61	62	70
Speed-up to GAP	1	181	178	158

Supplementary Figures.

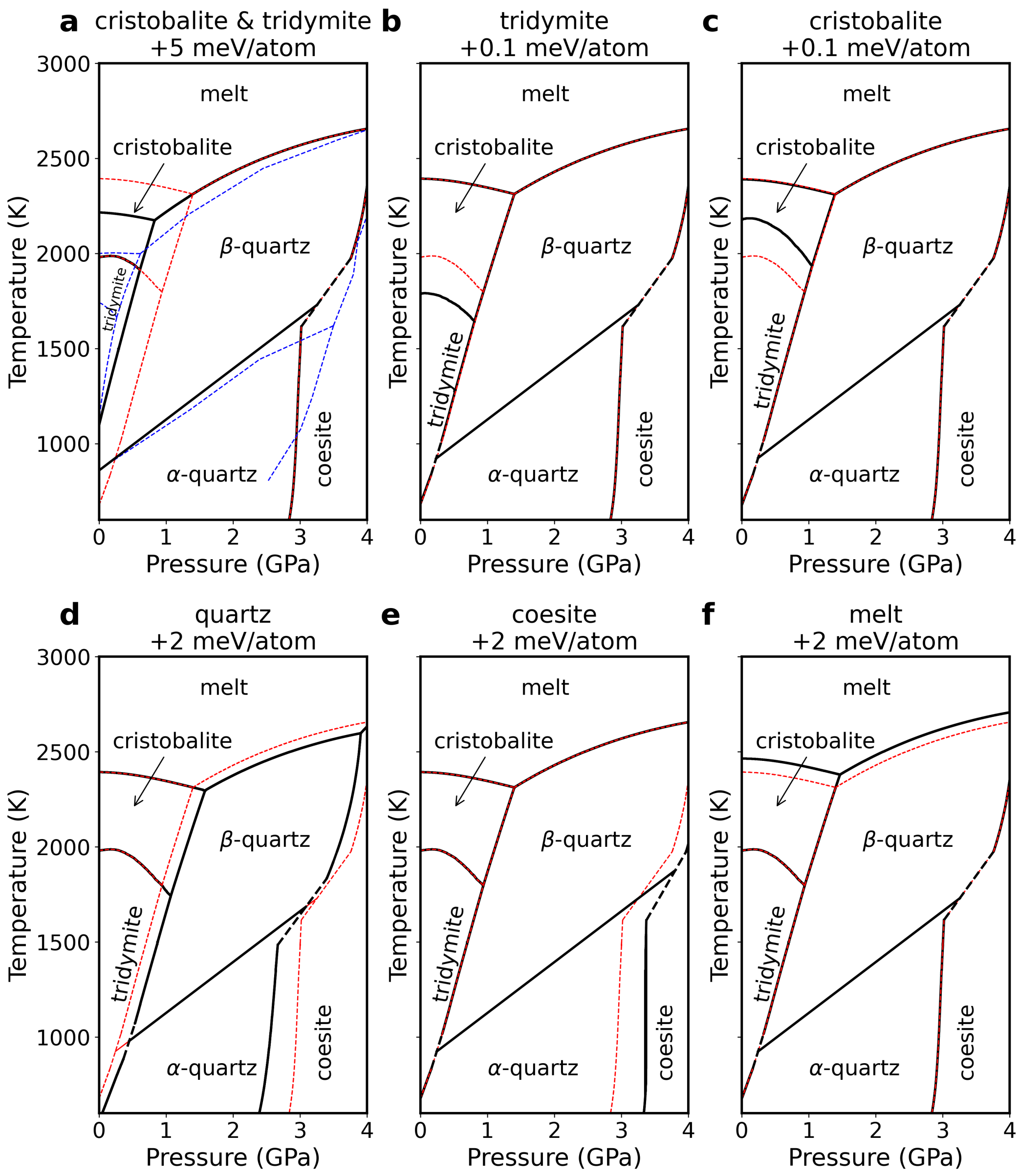

Supplementary Figure 1. **Uncertainty of the phase diagram.** Modified phase diagrams are shown in which we made certain phases less stable by adding a penalty term to the free energies obtained using the ACE potential. The red lines in the background correspond to the native phase diagram. Panel (a) shows the phase diagram for the case that cristobalite and tridymite are 5 meV/atom less favourable. The blue lines correspond to the CALPHAD phase diagram.[S9] Panels (b) and (c) show the phase diagram that would result if tridymite or cristobalite had a 0.1 meV/atom higher energy. Similarly, panels (d–f) show the phase diagram that would result if quartz, coesite, or the melt had a 2 meV/atom higher energy, respectively.

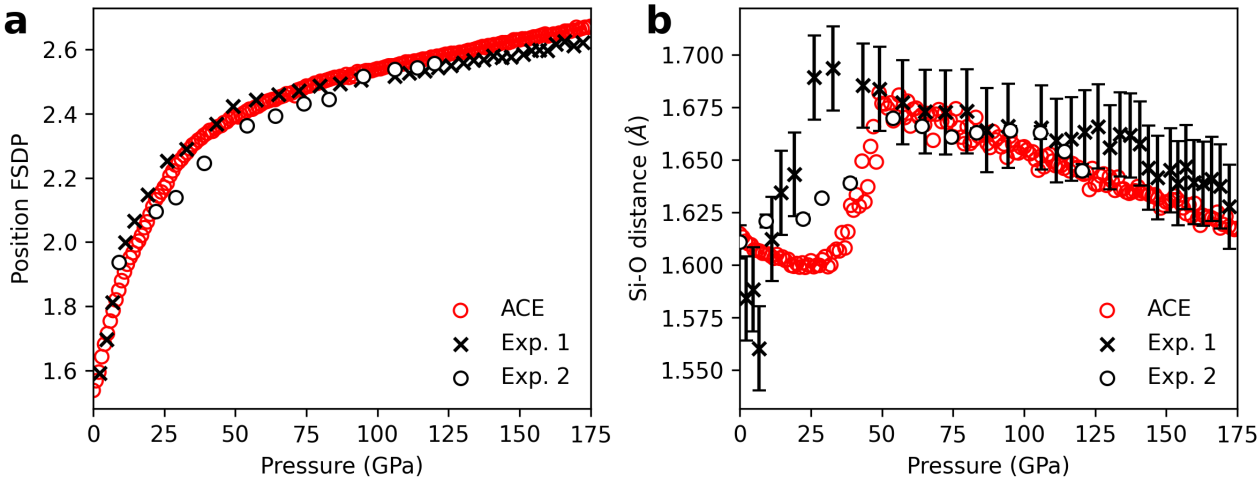

Supplementary Figure 2. **Structural fingerprints of amorphous silica under high-pressure.** (a) Position of the first sharp diffraction peak (FSDP) of the structure factor for various pressures predicted by the atomic cluster expansion (ACE). (b) Position of the first peak of the Si–O radial distribution function, indicating the typical Si–O bond distance. The experimental values are from Refs. 14 and 15.

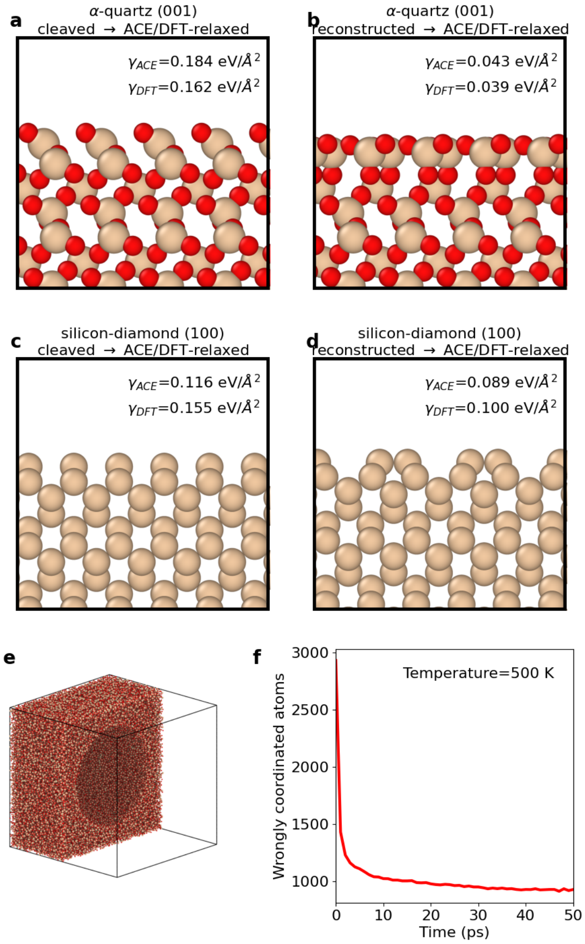

Supplementary Figure 3. **Reconstruction of various surfaces.** (a) The cleaved α-quartz (001) surface and the corresponding surface energies. Here and in the following γ_{ACE} corresponds to the surface energy predicted by the atomic cluster expansion and γ_{DFT} corresponds to the surface energy predicted by density functional theory. (b) The stable reconstruction from Ref. 16 and the corresponding surface energies, which are significantly lower than that of the cleaved surface. (c) The (100) surface of diamond-type silicon, with the surface energy indicated. (d) Same for the p(2×1) reconstruction of the (100) surface, which is more stable than the cleaved surface. (e) Sliced view of an amorphous silica structure in which a pore was cut manually. The surface inside has a large number of wrongly coordinated atoms. (f) Upon annealing this structure at 500 K, the number of wrongly-coordinated atoms steadily decreases over time, indicating surface reconstructions.

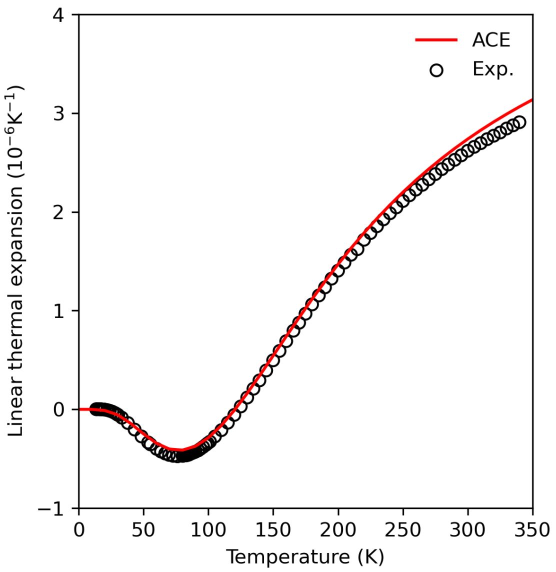

Supplementary Figure 4. **Linear thermal expansion coefficient of diamond** calculated with the atomic cluster expansion (ACE) potential, using the quasi-harmonic approximation[S10] as implemented in `phonopy` [S11,12] compared to experimental values from Ref. 17.

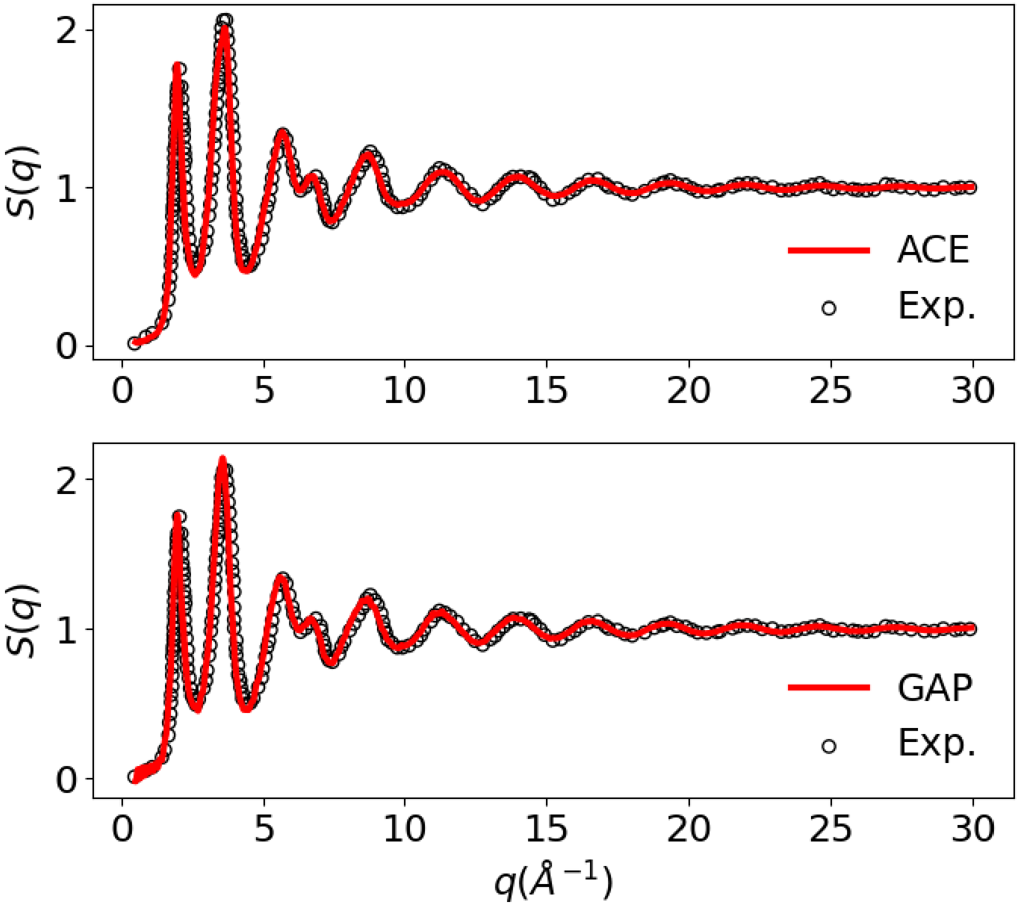

Supplementary Figure 5. **Structure factor of amorphous silicon.** The structure factor $S(q)$ as a functions of the wave vector q of an amorphous silicon structure generated with the atomic cluster expansion (ACE) potential is compared to the experimentally determined structure factor from Ref. 18 and the structure factor of an 4,096-atom amorphous silicon structure generated using the Gaussian approximation potential for silicon (GAP) model (lower panel), taken from Ref. 13. The latter structure was equilibrated at 300 K with the GAP to determine the structure factor. We note that slight discrepancies can be caused by different sizes of the structures.

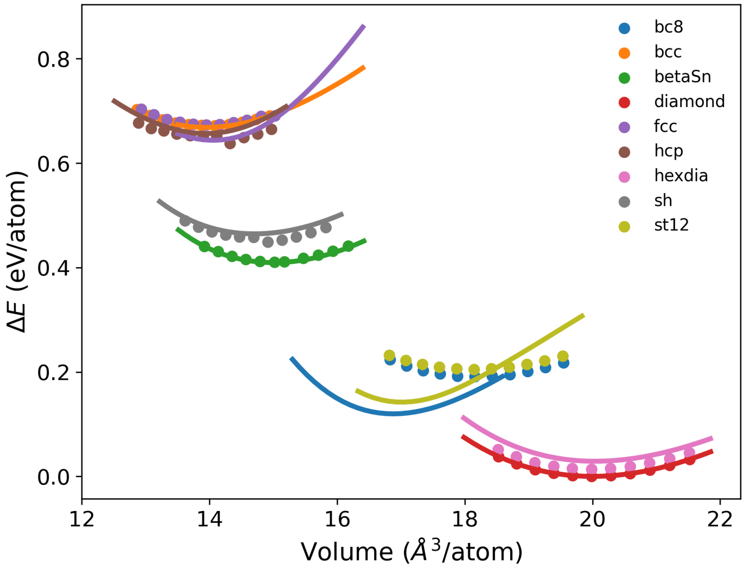

Supplementary Figure 6. **Hydrostatic energy–volume curves of various silicon modifications.** The dots indicate the relaxed density functional theory (DFT) energies for each modification, whereas the lines are the energy–volume curves predicted by the atomic cluster expansion (ACE) potential. ΔE is the energy difference reference to the ground state, which is the silicon diamond structure.

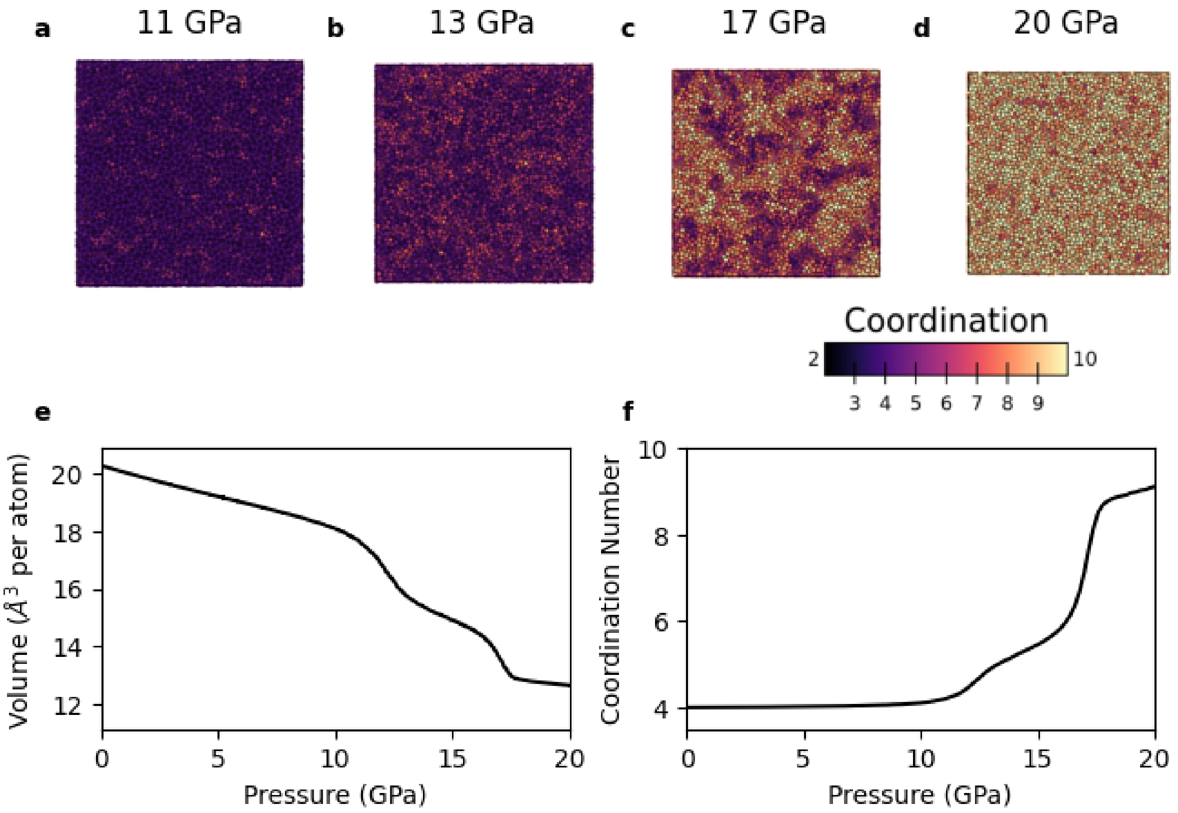

Supplementary Figure 7. **Polyamorphic transition of silicon.** Panels (a–d) show snapshots of amorphous silicon under various pressures during an isostatic compression run. Panels (e) and (f) show the volume per atom and the coordination number, respectively, dependent on the pressure for the same simulation. We used the same simulation protocol as in Ref. 19.

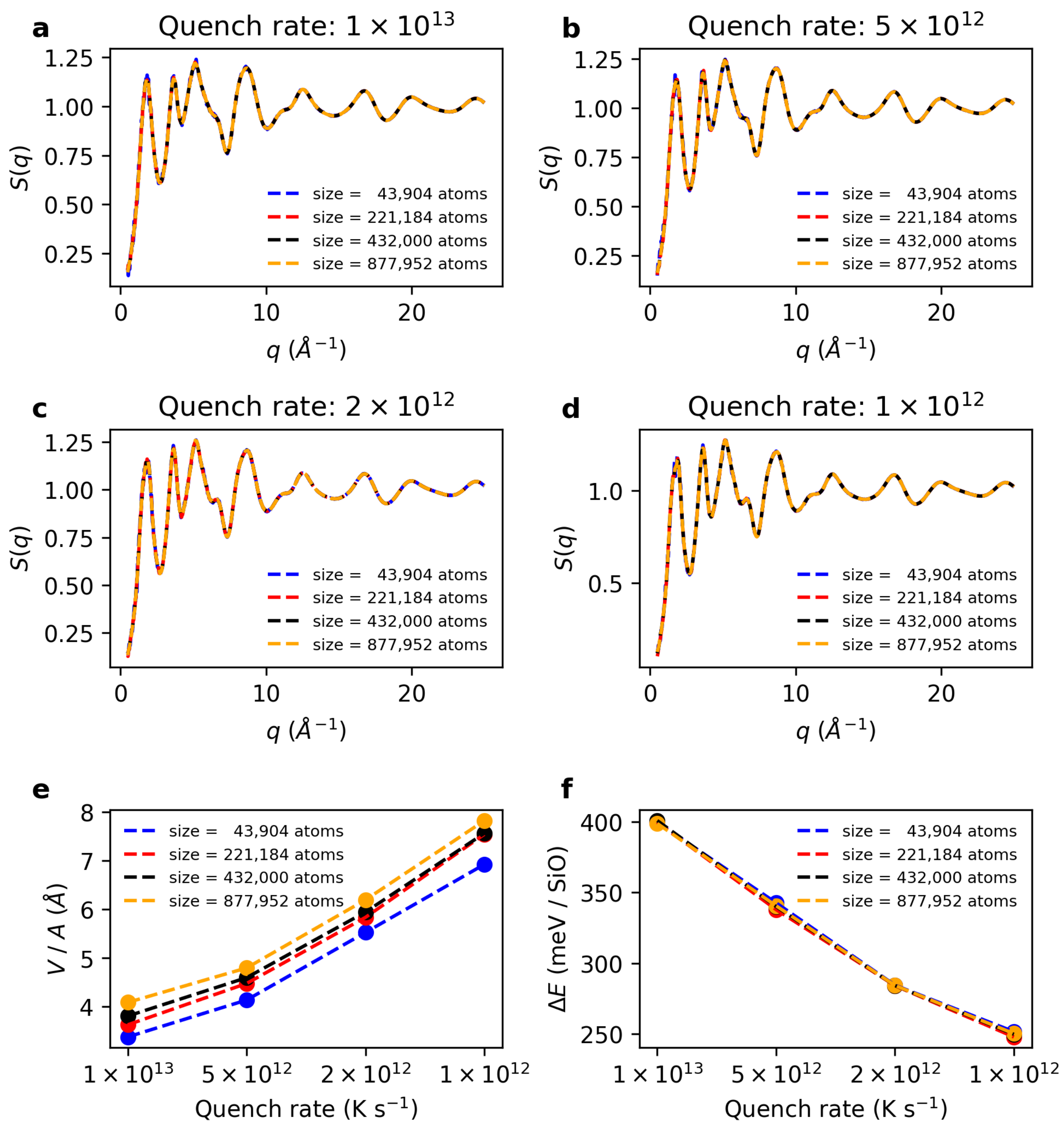

Supplementary Figure 8. **System-size dependence of properties of the SiO structure.** In panels (a–d), we show the system-size dependence of the structure factor $S(q)$ as function of the wave vector q of SiO structures for different quench rates. In panel (e), we show the ratio between interface area A and grain volume V of the silicon grains for different system sizes and quench rates. In panel (f), we show the same for the energy of formation ΔE referenced to α-quartz and silicon (diamond-type).

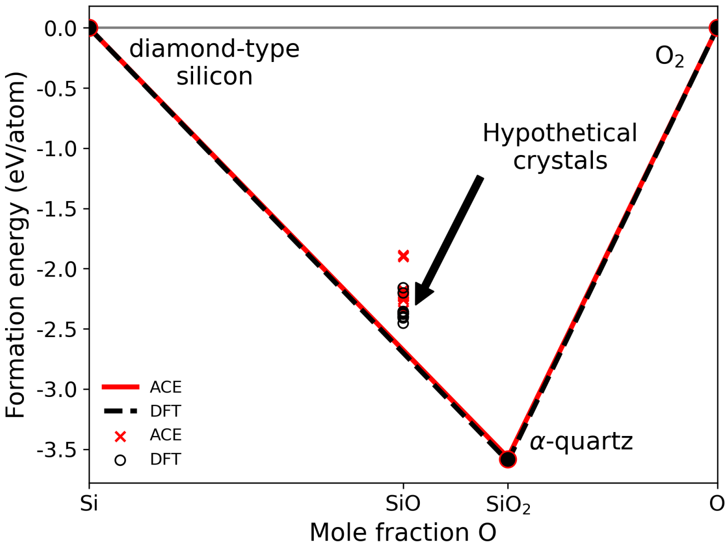

Supplementary Figure 9. **Convex hull of the Si–O system.** For the stochiometric composition SiO, we added the formation energies of the hypothetical ambient-pressure crystal structures from Ref. 20. The energies of these structures as predicted by the atomic cluster expansion (ACE) are marked in red, while the density functional theory (DFT) results are marked in black. None of these hypothetical structures have been included in the training database.

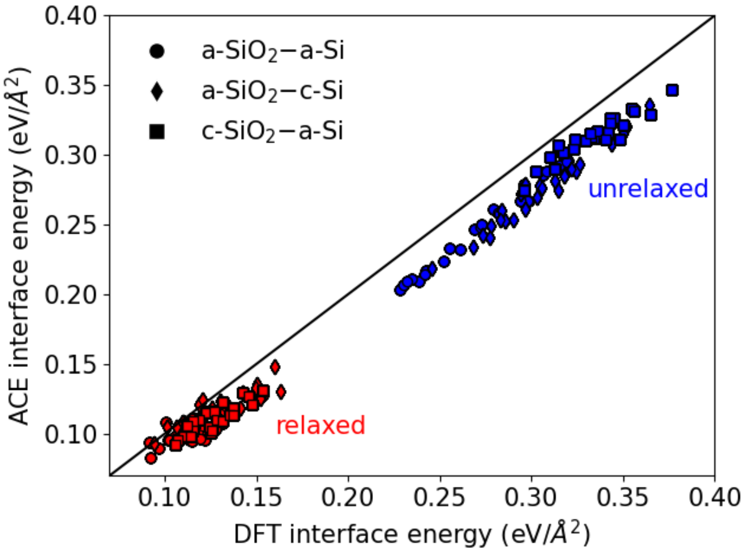

Supplementary Figure 10. **Interface energies between silicon and silica.** Various interfaces between amorphous silica and amorphous silicon, and amorphous silica and crystalline silicon (diamond), as well as crystalline silica (α-quartz) and amorphous silicon have been constructed. The interface energies are shown for unrelaxed (blue) and relaxed (red) configurations, comparing the atomic cluster expansion (ACE) potential predictions to density-functional theory (DFT) data. To obtain DFT energies, we used single-point energy computations for the ACE-relaxed structures.

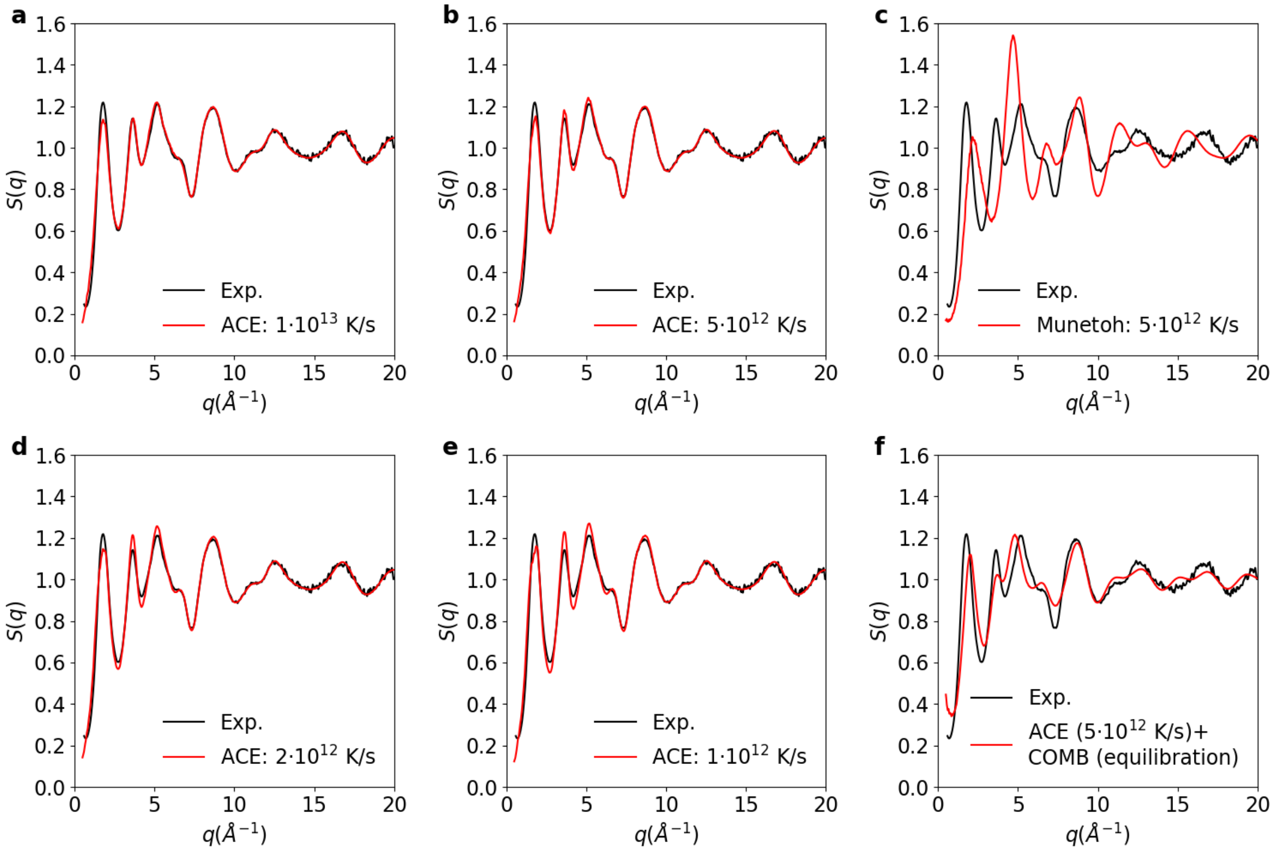

Supplementary Figure 11. **Structure factors of SiO.** Panels (a–b) and (d–e) show structure factors $S(q)$ as a function of the wave vector q of SiO model structures generated by different quench rates using the atomic cluster expansion (ACE) potential. The corresponding structure pictures are shown in the main text. Panel (c) shows the structure factor of a structure generated with the same protocol, but with the Munetoh potential.[S21] Panel (f) shows the structure factor of the structure from (b) after relaxation with the charge optimized many-body (COMB) potential for the Si–O system.[S22]

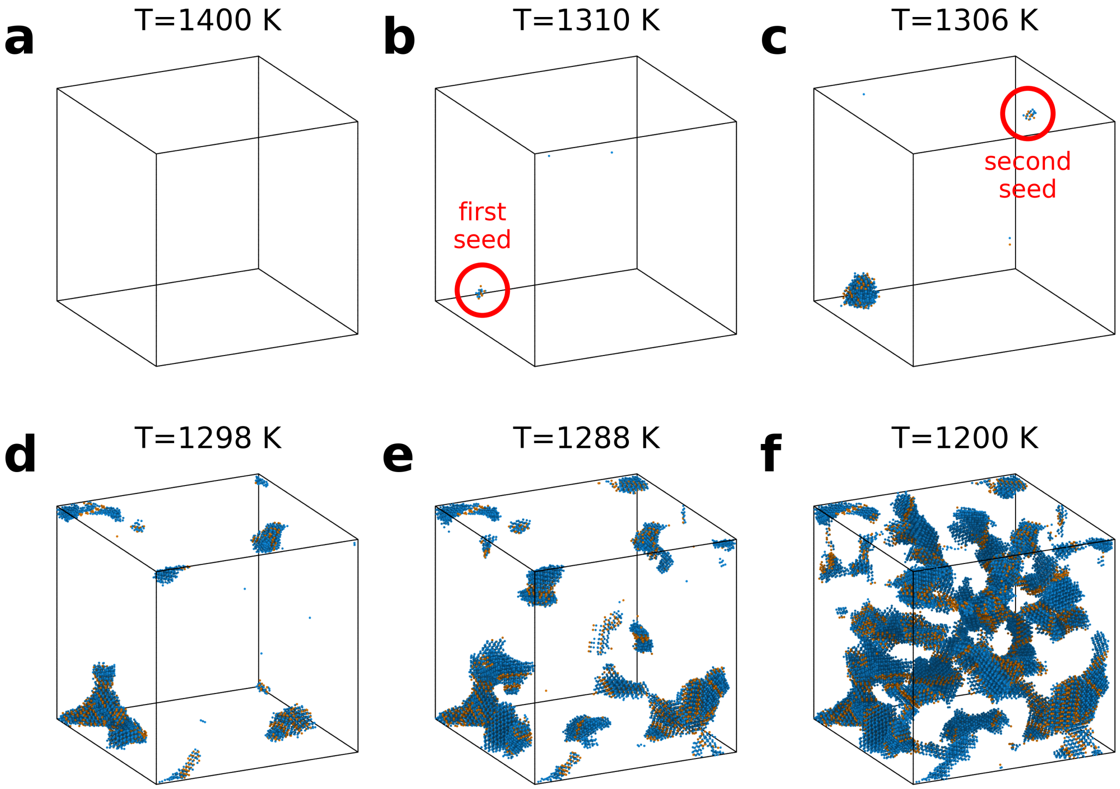

Supplementary Figure 12. **Crystallisation process of silicon in SiO.** Panels (a–f) show the "crystalline-silicon-like" atoms recognised by Polyhedral Template Matching (Root mean square deviation: 0.1) during a quench from 1,400 K to 1,200 K. The amorphous silicon as well as the amorphous silica part of the structure is not shown. At 1,310 K and 1,306 K, the first crystalline seeds appear. Both seeds strongly increase in size [panels (d–e)], until nearly the whole structure is permeated by crystalline silicon, as seen in panel (f).

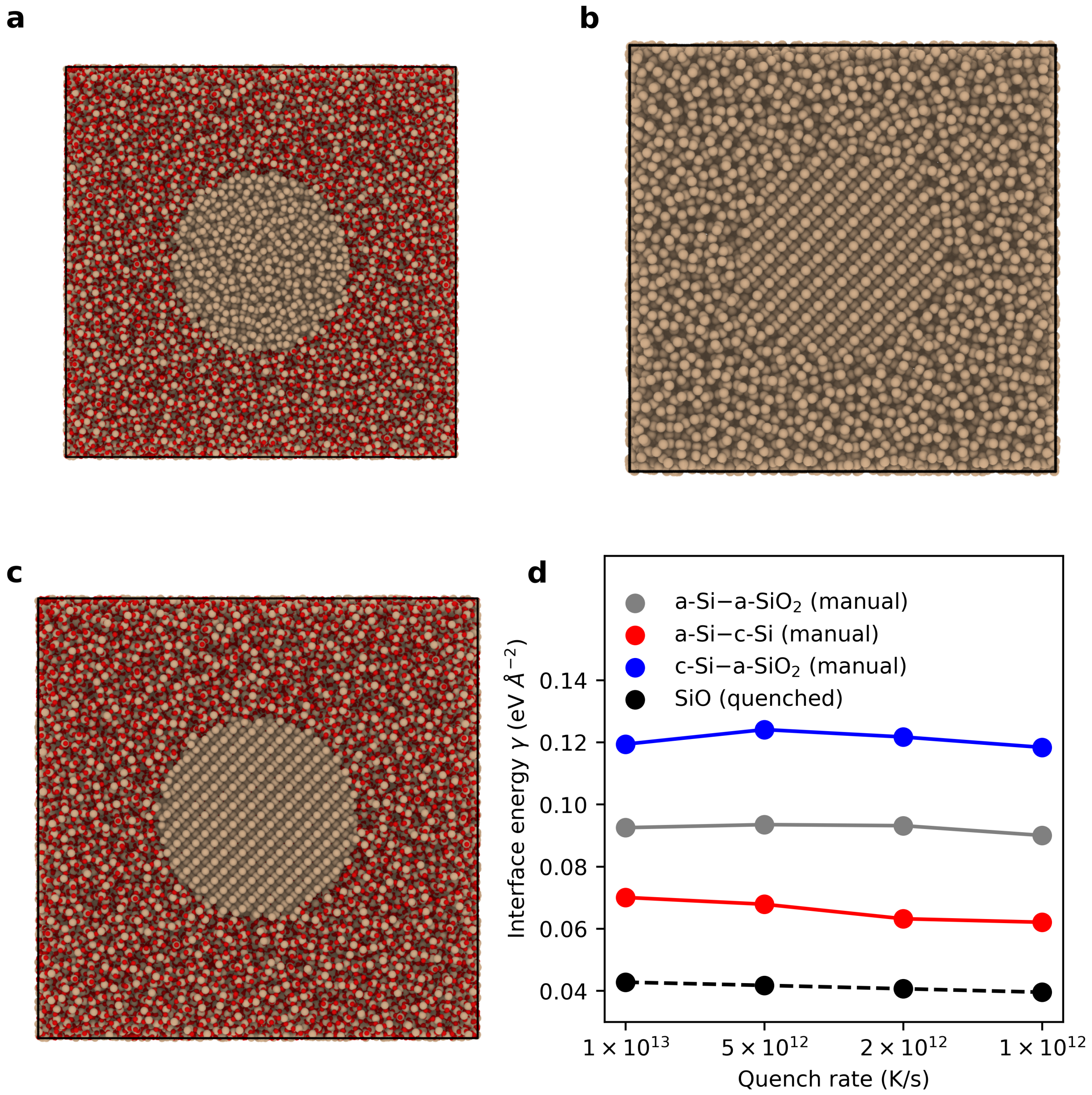

Supplementary Figure 13. **Interfaces between silicon and silica.** Panels (a–c) show various manually constructed interface structures with spherical inclusions. Panel (d) shows the interface energies of these structures. The quench rate on the x-axis corresponds to the amorphous input structures for the manually constructed structures and, in the case of the SiO structures, to the quench rate with which the mixed structures were quenched.

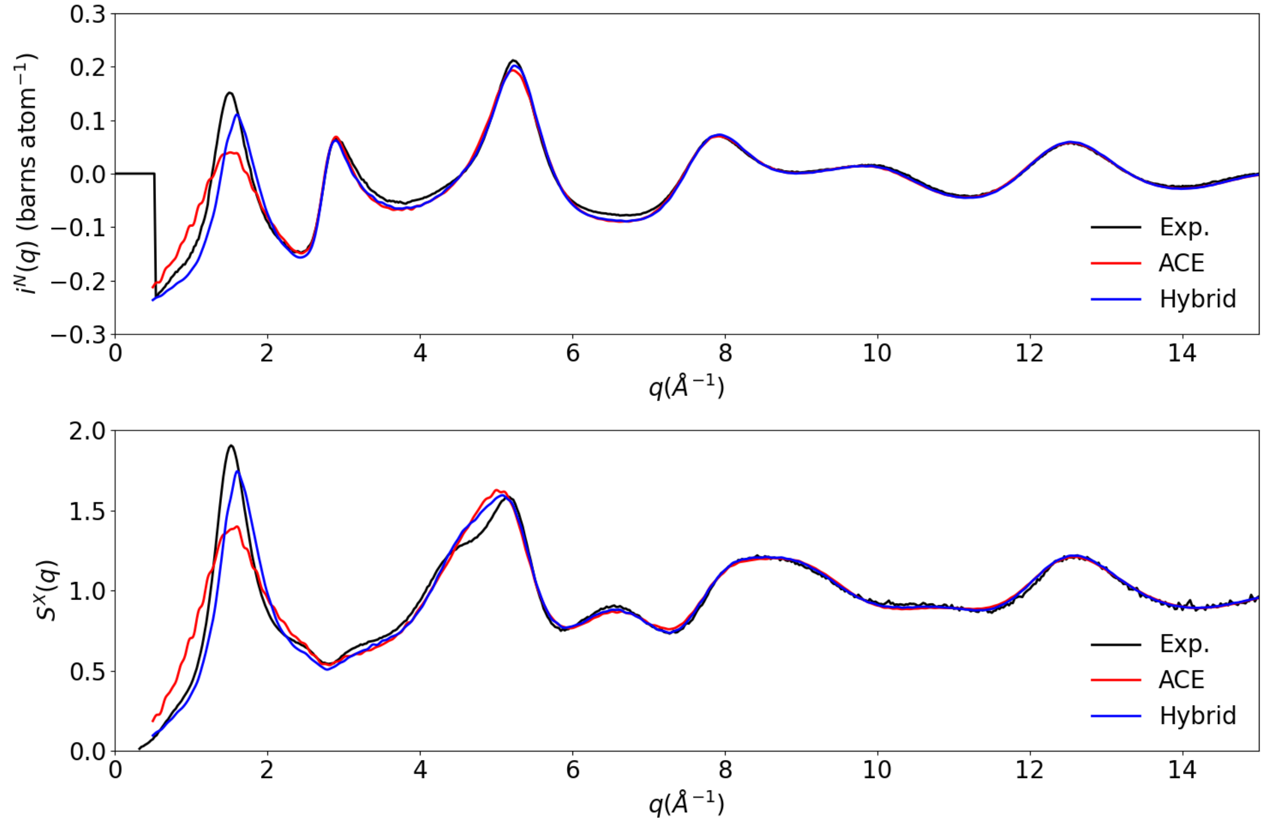

Supplementary Figure 14. **Neutron and x-ray structure factors of amorphous silica.** The upper panel shows the distinct neutron scattering $i^N(q)$ while the lower panel shows the x-ray structure factor $S(q)$ both as function of the wave vector q. Models have been generated using the ACE potential (quench rate: 10^{12} K/s) and by a hybrid protocol using the CHIK[S23] and atomic cluster expansion (ACE) potential following the approach in Ref. 1 (quench rate: 10^{11} K/s). The neutron diffraction data are taken from Ref. 24 and the x-ray diffraction data are taken from Ref. 25. The cut-off used for the determination of the radial distribution functions was set to 60 Å.

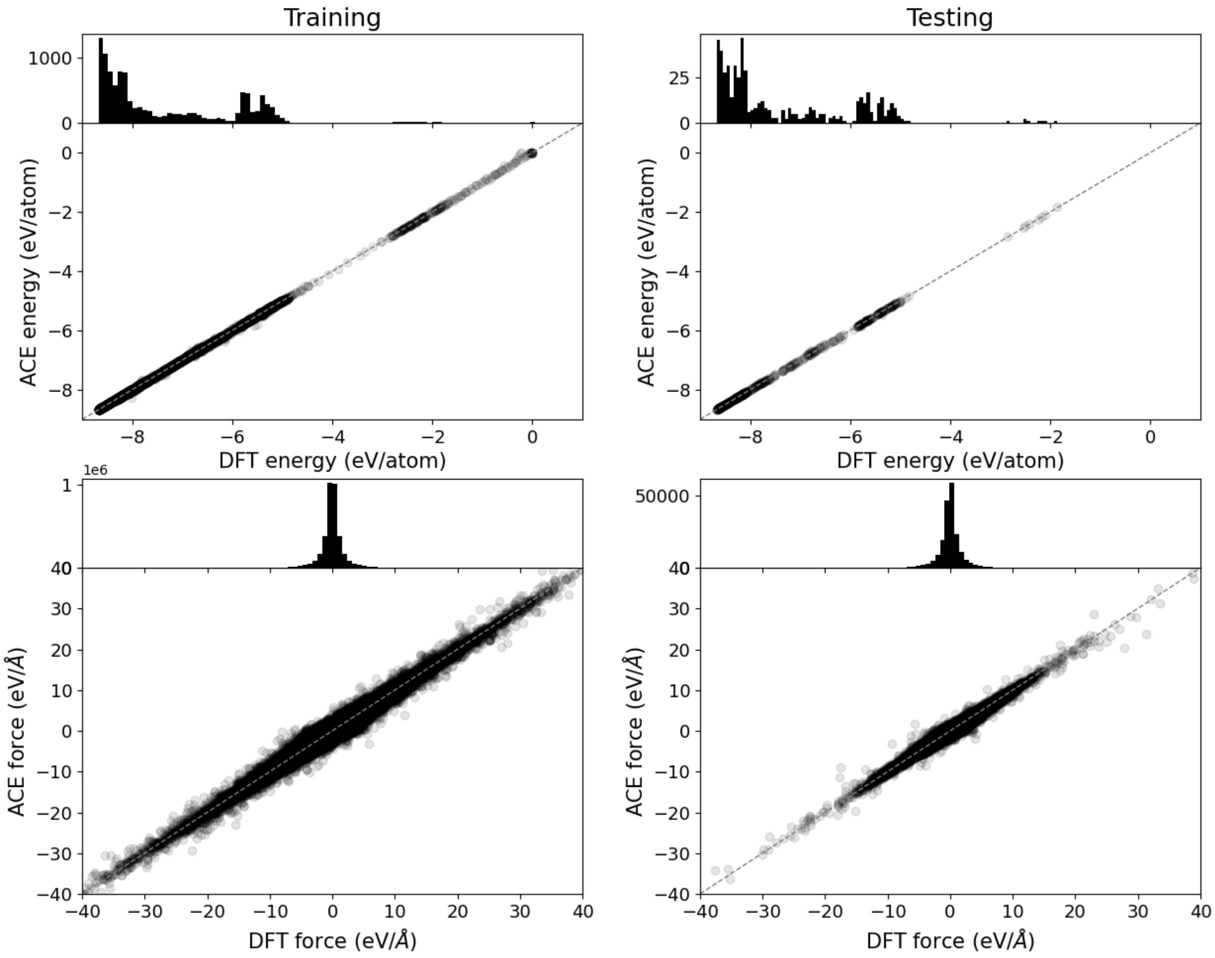

Supplementary Figure 15. **Scatter plots** for the energies (*top*) and forces (*bottom*) of the training and testing set. The total dataset was randomly divided into the training (95%) and the testing set (5%). Above the scatter plots, we show the distribution of data points. The energies and forces are calculated by the atomic cluster expansion potential (ACE) and density functional theory (DFT).

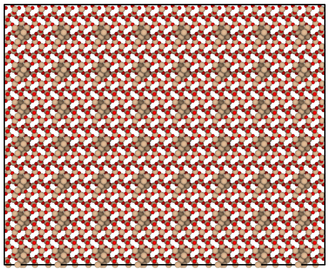

Supplementary Figure 16. **Initial structure for the large-scale SiO_x interpolation models.** The structural model is a supercell of α-cristobalite structures merged with diamond-type silicon structures.

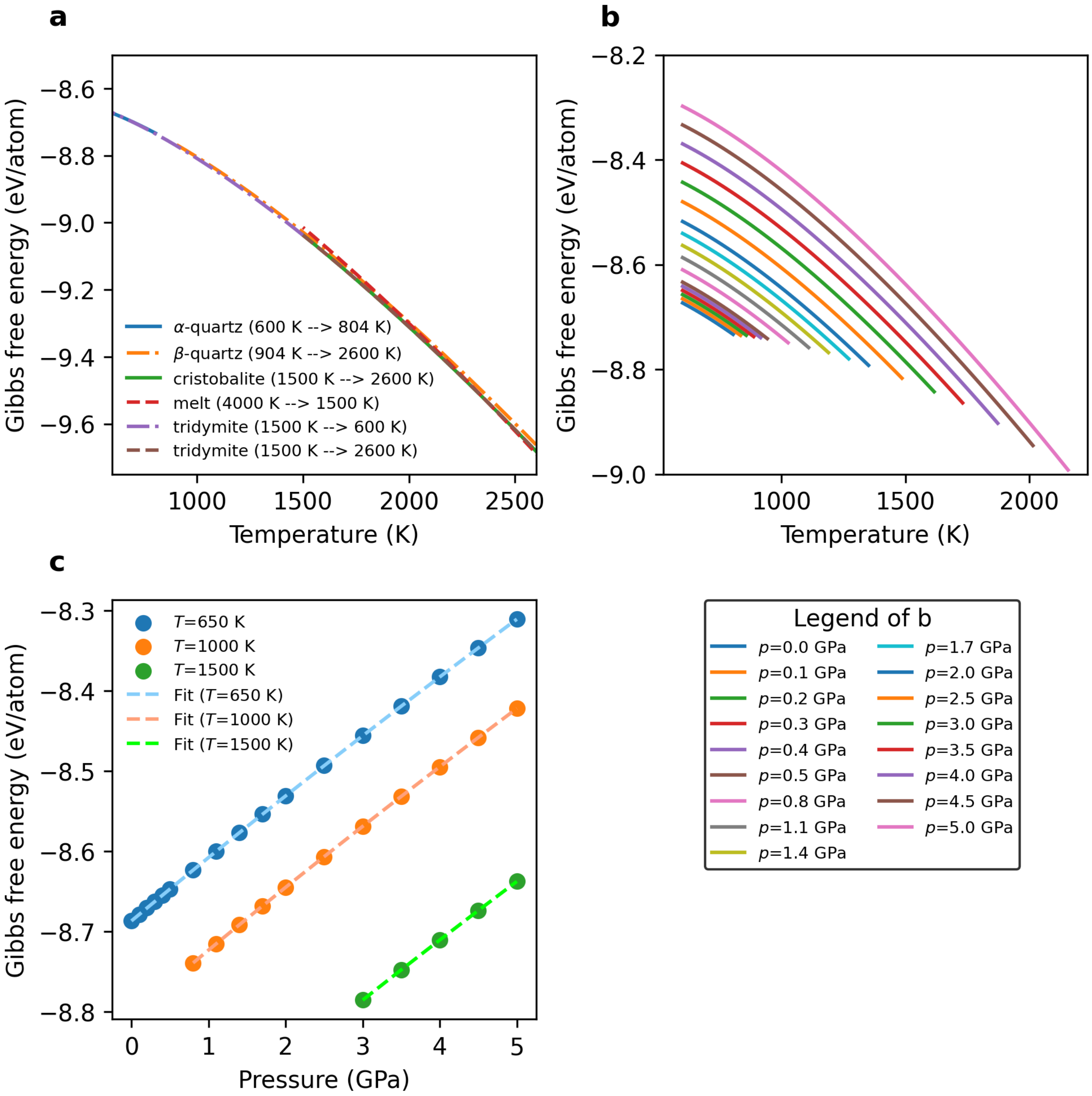

Supplementary Figure 17. **Free energies by thermodynamic integration.** (a) Gibbs free energies calculated by `calphy` for various silica polymorphs for different temperature ranges at 0 GPa pressure. (b) Gibbs free energy results for α-quartz at various pressures. (c) Polynomial fits of the Gibbs free energy of α-quartz as dependent on the pressure at different temperatures.

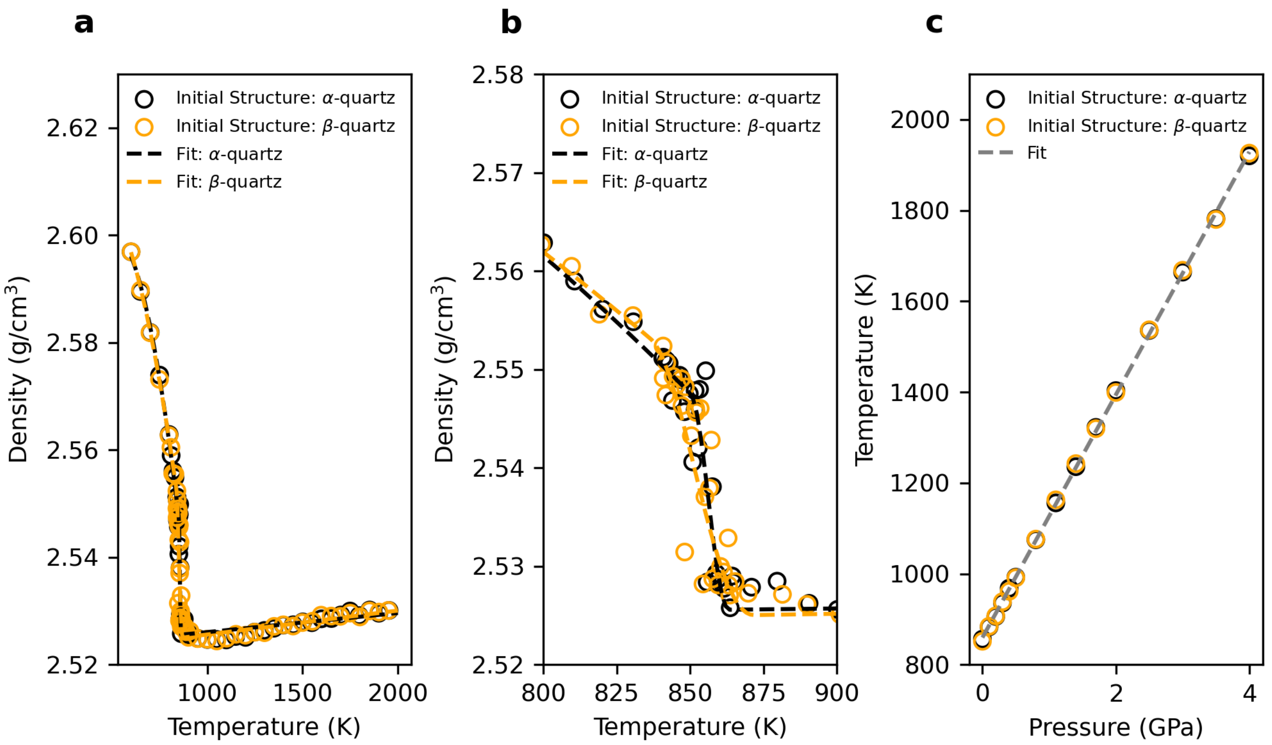

Supplementary Figure 18. **Determination of the α-/β-quartz transition.** (a) Density of quartz depending on the temperature for a pressure of 0 GPa. It can be seen that there is a distinct transition from α-quartz to β-quartz. (b) The same data as in (a) but limited to a temperature range of 800-900 K. In this plot, it can be seen that the transition from α- to β-quartz appears around 850 K. Moreover, we see that the fitting functions agree with the data. Panel (c) shows the transition temperature from α-quartz to β-quartz as dependent on the pressure.

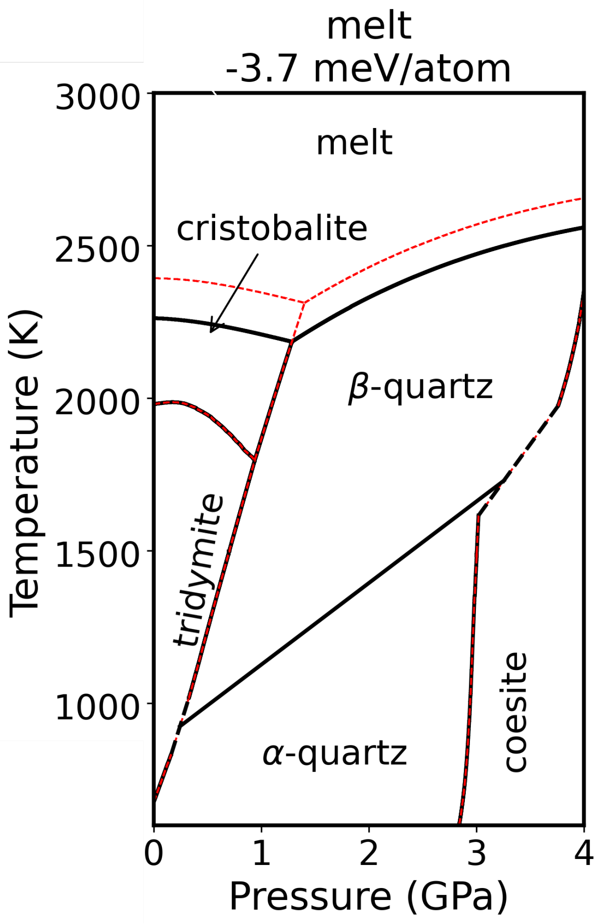

Supplementary Figure 19. **Modified phase diagram with a lower-energy liquid phase.** The red lines show the phase diagram as predicted by the atomic cluster expansion (ACE) potential, while the black lines correspond to the phase diagram under the assumption that the liquid phase is more favourable by 3.7 meV/atom than predicted by the ACE potential.

Supplementary References.

[S1] Erhard, L. C., Rohrer, J., Albe, K. & Deringer, V. L. A machine-learned interatomic potential for silica and its relation to empirical models. *npj Computational Materials* **8**, 1–12 (2022).

[S2] Bartók, A. P., Kermode, J., Bernstein, N. & Csányi, G. Machine Learning a General-Purpose Interatomic Potential for Silicon. *Physical Review X* **8**, 041048 (2018).

[S3] Novikov, I. S., Gubaev, K., Podryabinkin, E. V. & Shapeev, A. V. The MLIP package: Moment tensor potentials with MPI and active learning. *Machine Learning: Science and Technology* **2**, 025002 (2020).

[S4] Larsen, A. H. *et al.* The atomic simulation environment—a Python library for working with atoms. *J. Phys.: Condens. Matter* **29**, 273002 (2017).

[S5] Himanen, L. *et al.* DScribe: Library of descriptors for machine learning in materials science. *Computer Physics Communications* **247**, 106949 (2020).

[S6] Menon, S., Lysogorskiy, Y., Rogal, J. & Drautz, R. Automated free-energy calculation from atomistic simulations. *Physical Review Materials* **5**, 103801 (2021).

[S7] de Koning, M., Antonelli, A. & Yip, S. Optimized Free-Energy Evaluation Using a Single Reversible-Scaling Simulation. *Physical Review Letters* **83**, 3973–3977 (1999).

[S8] Paula Leite, R., Freitas, R., Azevedo, R. & de Koning, M. The Uhlenbeck-Ford model: Exact virial coefficients and application as a reference system in fluid-phase free-energy calculations. *The Journal of Chemical Physics* **145**, 194101 (2016).

[S9] Swamy, V., Saxena, S. K., Sundman, B. & Zhang, J. A thermodynamic assessment of silica phase diagram. *Journal of Geophysical Research: Solid Earth* **99**, 11787–11794 (1994).

[S10] Togo, A., Chaput, L., Tanaka, I. & Hug, G. First-principles phonon calculations of thermal expansion in Ti 3 SiC 2 , Ti 3 AlC 2 , and Ti 3 GeC 2. *Physical Review B* **81**, 174301 (2010).

[S11] Togo, A. & Tanaka, I. First principles phonon calculations in materials science. *Scripta Materialia* **108**, 1–5 (2015).

[S12] Togo, A. First-principles Phonon Calculations with Phonopy and Phono3py. *Journal of the Physical Society of Japan* **92**, 012001 (2023).

[S13] Deringer, V. L. *et al.* Realistic Atomistic Structure of Amorphous Silicon from Machine-Learning-Driven Molecular Dynamics. *J. Phys. Chem. Lett.* **9**, 2879–2885 (2018).

[S14] Prescher, C. *et al.* Beyond sixfold coordinated Si in SiO $_2$ glass at ultrahigh pressures. *Proceedings of the National Academy of Sciences* **114**, 10041–10046 (2017).

[S15] Kono, Y., Shu, Y., Kenney-Benson, C., Wang, Y. & Shen, G. Structural Evolution of SiO_2 Glass with Si Coordination Number Greater than 6. *Physical Review Letters* **125**, 205701 (2020).

[S16] Goumans, T. P. M., Wander, A., Brown, W. A. & Catlow, C. R. A. Structure and stability of the (001) α-quartz surface. *Physical Chemistry Chemical Physics* **9**, 2146–2152 (2007).

[S17] Lyon, K. G., Salinger, G. L., Swenson, C. A. & White, G. K. Linear thermal expansion measurements on silicon from 6 to 340 K. *Journal of Applied Physics* **48**, 865–868 (1977).

[S18] Laaziri, K. *et al.* High-energy x-ray diffraction study of pure amorphous silicon. *Physical Review B* **60**, 13520–13533 (1999).

[S19] Deringer, V. L. *et al.* Origins of structural and electronic transitions in disordered silicon. *Nature* **589**, 59–64 (2021).

[S20] AlKaabi, K., Prasad, D. L. V. K., Kroll, P., Ashcroft, N. W. & Hoffmann, R. Silicon Monoxide at 1 atm and Elevated Pressures: Crystalline or Amorphous? *Journal of the American Chemical Society* **136**, 3410–3423 (2014).

[S21] Munetoh, S., Motooka, T., Moriguchi, K. & Shintani, A. Interatomic potential for Si–O systems using Tersoff parameterization. *Computational Materials Science* **39**, 334–339 (2007).

[S22] Shan, T.-R. *et al.* Second-generation charge-optimized many-body potential for Si / SiO 2 and amorphous silica. *Physical Review B* **82**, 235302 (2010).

[S23] Carré, A., Horbach, J., Ispas, S. & Kob, W. New fitting scheme to obtain effective potential from Car-Parrinello molecular-dynamics simulations: Application to silica. *EPL (Europhysics Letters)* **82**, 17001 (2008).

[S24] ISIS Disordered Materials Database, http://www.alexhannon.co.uk/.

[S25] Mei, Q., Benmore, C. J. & Weber, J. K. R. Structure of liquid SiO_2: A Measurement by High-Energy X-Ray Diffraction. *Physical Review Letters* **98**, 057802 (2007).
